# Opposing aftereffects between a White male face set and a diverse face set

**DOI:** 10.1177/03010066221132470

**Published:** 2022-11-16

**Authors:** Victoria Foglia, M.D. Rutherford

**Affiliations:** McMaster University, Canada

**Keywords:** aftereffects, diversity, face perception, opposing aftereffects

## Abstract

Opposing aftereffects have been observed for faces categorized by gender, race, and age. In order to form opposing aftereffects, it appears that the two face sets must be both physically distinct and differ in terms of social meaning. The current study tests whether (1) a face set that is diverse with respect to sex and race can produce a coherent aftereffect and (2) whether this diversity itself is socially meaningful enough to support opposing aftereffects. Participants adapted to a homogenous face set consisting of only White male Republican congressmen and a diverse face set consisting of White, Asian, Black, and Latino male and female Democratic congress members. Opposing aftereffects were observed: participants adapted simultaneously and in opposite directions to the face sets. These results are the first evidence of adaptation to a face set that varies based on race and sex, and the first evidence of diversity being perceived as a socially meaningful category marker.

Aftereffects have been called the “psychologists’ microelectrode” for their ability to reveal how stimuli are represented in the brain (Frisby, 1979). A visual aftereffect is a short-term change to the perception of a stimulus after fixating on the adapting stimulus, as when a white surface appears green after a period of fixating on a red stimulus. The process of adaptation temporarily alters the perceptual system (Clifford & Rhodes, 2005; Webster, 2011). A classic example is the waterfall illusion, in which observers adapt to a waterfall that is moving downward, then view a stationary image of a waterfall that appears to move upwards (Mather et al., 1998). Visual aftereffects have been found for orientation (Clifford, 2002), color (Gurnsey et al., 1994; Thompson & Latchford, 1986), size (Blakemore & Sutton, 1969; Sutherland, 1961), and shape (Suzuki, 2003).

Visual aftereffects have been observed using complex stimuli such as faces, as when the face has been experimentally altered (Leopold et al., 2005; Watson & Clifford, 2003; Webster & Maclin, 1999). For example, after continuously viewing faces with digitally contracted face features, an unaltered face will appear expanded (Leopold et al., 2001). This type of face adaptation is a simple aftereffect (Jaquet & Rhodes, 2008). Simple aftereffects have been observed for identity (Leopold et al., 2001; Rhodes & Jeffery, 2006; Rhodes & Leopold, 2011), attractiveness (Rhodes et al., 2003), race (Webster et al., 2004), gender (Rhodes et al., 2004; Webster et al., 2004), and emotion (Webster et al., 2004), and are taken as evidence of norm-based coding (Rhodes et al., 2003; Rhodes & Leopold, 2011; Webster et al., 2004).

In opposing aftereffects experiments, participants are adapted simultaneously to two separate face categories that have been physically distorted in the opposite directions, for example, male contracted and female expanded faces (Jaquet & Rhodes, 2008; Jaquet et al., 2008; Little et al., 2008; Rhodes et al., 2004). If attractiveness or normality selections shift in the direction of adaptation for both categories, opposing aftereffects have been found. Opposing aftereffects are taken as evidence that the face categories are processed using discrete neural representations as an adaptation to one face category does not interfere with the adaptation or representation of another face category (Jaquet et al., 2008). Opposing aftereffects have been observed for race (Jaquet et al., 2008; Little et al., 2008), gender (Jaquet et al., 2008; Little et al., 2005), age (Little *et al*., 2008), and species (Little et al., 2008).

There are two criteria that appear to be prerequisites in order to evoke opposing aftereffects, (1) the two face sets must be physically distinct, and (2) the face sets must be socially meaningful. Short and Mondloch (2010) tested whether opposing aftereffects could occur between two face sets without physical differences. Artificial “in groups” or “out-groups” based on purported shared personality traits were created to form a meaningful social distinction while maintaining physical similarity between the two face sets. Opposing aftereffects were not observed, suggesting that face categories must be physically different as well as socially meaningful in order to evoke opposing aftereffects. In contrast, Bestelmeyer et al. (2008) examined whether physical differences could evoke opposing aftereffects in the absence of a socially meaningful category distinction by controlling for physical dissimilarity across a socially distinct and a less socially distinct pair of face sets. Opposing aftereffects were observed across the male and female face sets but not across the female and hyper-female face sets, despite the fact that the physical dissimilarity was equated. These results suggested that the female and hyper-female face sets were not socially distinct in the manner that supports opposing aftereffects, while the male and female sets were.

While it is believed that face sets must be different with respect to social meaning, there has been very little work exploring what constitutes a socially meaningful distinction, although we know that both sex and race support opposing aftereffects. It is possible that the diversity of a face set, with respect to sex and race, may be a meaningful cue to social category membership, if the diversity itself serves as a cue to a cultural competence among group members. For example, in the United States, members of the Democratic party are more ethnically diverse, and party leadership has more gender diversity compared to the Republican party. Thus, the perceived diversity of the two groups could be a cue used to categorize them as Democrats or Republicans. Commonly, opposing aftereffect experiments consist of two distinct, but homogeneous face sets (i.e., male/female, Asian/White, Human/Ape (Bestelmeyer et al., 2008; Jaquet & Rhodes, 2008; Little et al., 2008). Previously no examination of race contingent aftereffects (Armann et al., 2011; Jaquet et al., 2008; Little et al., 2008) has varied the races of the individuals within an adapting face set and have only compared whether it is possible to adapt to two separate race categories at one time. Little is known about how the diversity of a face set may affect aftereffects, how diversity is perceived categorically, and whether it can be perceived as one uniform social group.

## Current Study

The current study is designed to test (1) whether it is possible to adapt to a category of faces that vary physically within a group and (2) whether diversity itself is a cue to category membership and thus supports the formation of face adaptation. Using an opposing aftereffects paradigm, participants in the current study adapted to either a contracted diverse face set or expanded homogenous face set or to an expanded diverse face set and contracted homogenous face set. Evidence of opposing aftereffects would indicate differences in adaptation to the two face sets. Participants who adapt to contracted diverse and expanded homogenous faces are expected to prefer more contracted diverse faces (but fewer contracted homogenous faces) after adaptation.

Diverse and homogenous face sets depicted elected members of the United States. House of Representatives as of 2019. The homogenous face sets consist only of White male Republican Members of the House of Representatives. The diverse face set is a balanced sample of White, Latino, Black, and Asian men and women. These face sets were selected as they parallel the largely homogeneous Republican party and the relatively diverse Democratic party. The proportion of White men in the Democratic caucus is 38%, whereas the proportion of White men in the Republican caucus is 90% (Jacobs, 2018). Fifty-two of the Black House Members are Democrats, and one is Republican, 37 of all Latino or Hispanic members are Democrats and eight are Republican and 16 of all Asian representatives are Democratic while one is Republican (Manning, 2019). With respect to gender, 90 of the 105 female house members belong to the Democratic party, and the remaining 15 are Republican (Manning, 2019). Given this demographic distinction between the two parties, it is possible that diversity itself may be a cue to party membership. This is the first test of whether it is possible to adapt to a face set that varies in terms of physical characteristics. It is also the first examination as to how diverse face groups are perceived categorically in an opposing aftereffects paradigm.

## Methods

### Materials

#### Visual Stimuli

Forty-eight official portraits of current members of the United States Congress were obtained from a Wikipedia list of current congress members serving in the House of Representatives on June 17, 2019 (“List of Current Members of the United States House of Representatives,” 2019). From these portraits, a homogenous face set of 16 White Male Republicans and a diverse face set of 16 White, Latino, Black, and Asian male and female Democrats were selected. The diverse face set included four each of White, Latino, Black, and Asian faces, including two male and two female faces for each race. Images of Alexandria Ocasio-Cortez were excluded due to her notable presence in the media and on social media. Selecting by party was intended to maintain ecological validity. For a full list of the names of the Members of Congress used as visual stimuli see Appendix A.

**Figure 1.**
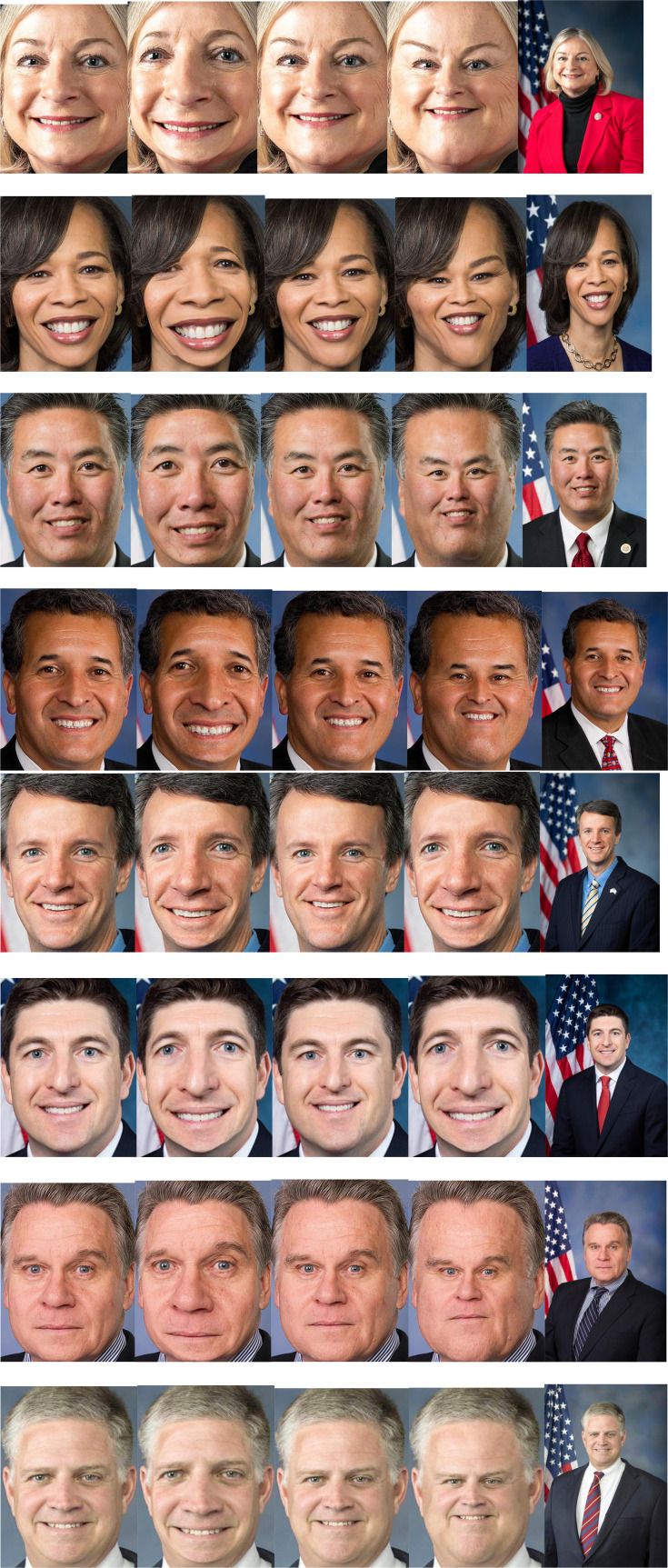
From left to right: 10% expanded, 60% expanded, 10% contracted, 60% contracted, and unaltered stimuli examples. Unaltered stimuli are only presented as a reference and were not displayed at any point in the experiment.

The faces were edited in Adobe Photoshop CS using a 4 by 5 aspect ratio so that images consisted of only the face, hair, and neck of each individual. The maximum resolution was maintained. Then each photo was edited to 3 inches by 4 inches with 300 pixels per inch. Finally, distortions were formed for each photo by expanding 10% and 60% and contracting 10% and 60% each using the spherize function including all the facial features. The 60% of distorted images were used as adapting stimuli in the adaptation phase. See Figure 1 for sample stimuli, unaltered, contracted, and expanded, at 10% and 60%.

#### Audio Stimuli

The audio stimuli to be played during pre-adaptation and post-adaptation or adaptation phases were recorded using the Voice Recorder Audio Editor cellphone application. The audio stimuli played during pre- and post-adaptation only stated the congressperson's name. The Audio recordings played during the adaptation phase stated the congressperson's name and the year when they entered office, for example, “Karen Bass entered office in 2017.” The purpose of the audio recordings was to socially engage participants in order to encourage a fulsome visual scan of the face without indicating which political party the faces may represent.

#### Procedure

Both of the adaptation conditions followed the same procedure but with opposite distortion during the adaptation trials. The procedure consisted of a pre-adaptation, an adaptation, and a post-adaptation phase. The audio stimuli were identical across conditions. The study was programmed and presented on E-Prime (Psychology Software Tools, Pittsburgh, PA). Each face image was presented on a 15-inch ASUS laptop with the participant at 25 inches away from the screen and at approximately 7° visual angle. Ethics approval was obtained from [Blinded] Research Ethics Board and informed consent were obtained from each participant before the experiment began.

#### Pre-Adaptation Phase

The purpose of this phase was to measure baseline face perception prior to any adaptation. Participants viewed a total of 64 face pairs for three seconds each. Each pair consisted of the same individual with one image expanded 10% and the other image contracted 10%. Pairs were displayed four times each, balancing the left–right placement of the expanded and contracted faces in a randomized order. These 64 trials comprised 16 face pairs. Eight face pairs were from the homogenous face set and eight faces were from the diverse face set. Each face pair was presented with an audio clip stating the congressperson's name.

Participants were asked to select which of the two faces was most attractive. Participants pressed the “F” key if the face on the left was more attractive and the “J” key if the face on the right was more attractive. Participants had unlimited time to respond, and once a selection was made, they were shown the next pair.

#### Adaptation Phase

Participants adapted to 32 faces, presented one at a time for 6 s each with an interstimulus interval of 500 ms. Each face was only presented once. Of the 32 faces, 16 were from the homogenous face set and 16 were from the diverse face set. The 60% expanded or contracted face stimuli were used during adaptation, based on the condition presented (e.g., 60% expanded homogeneous and contracted diverse or vice versa). The faces presented during adaptation were randomly selected at each trial from the set dictated by the condition.

#### Post-Adaptation Phase

**Figure 2.**
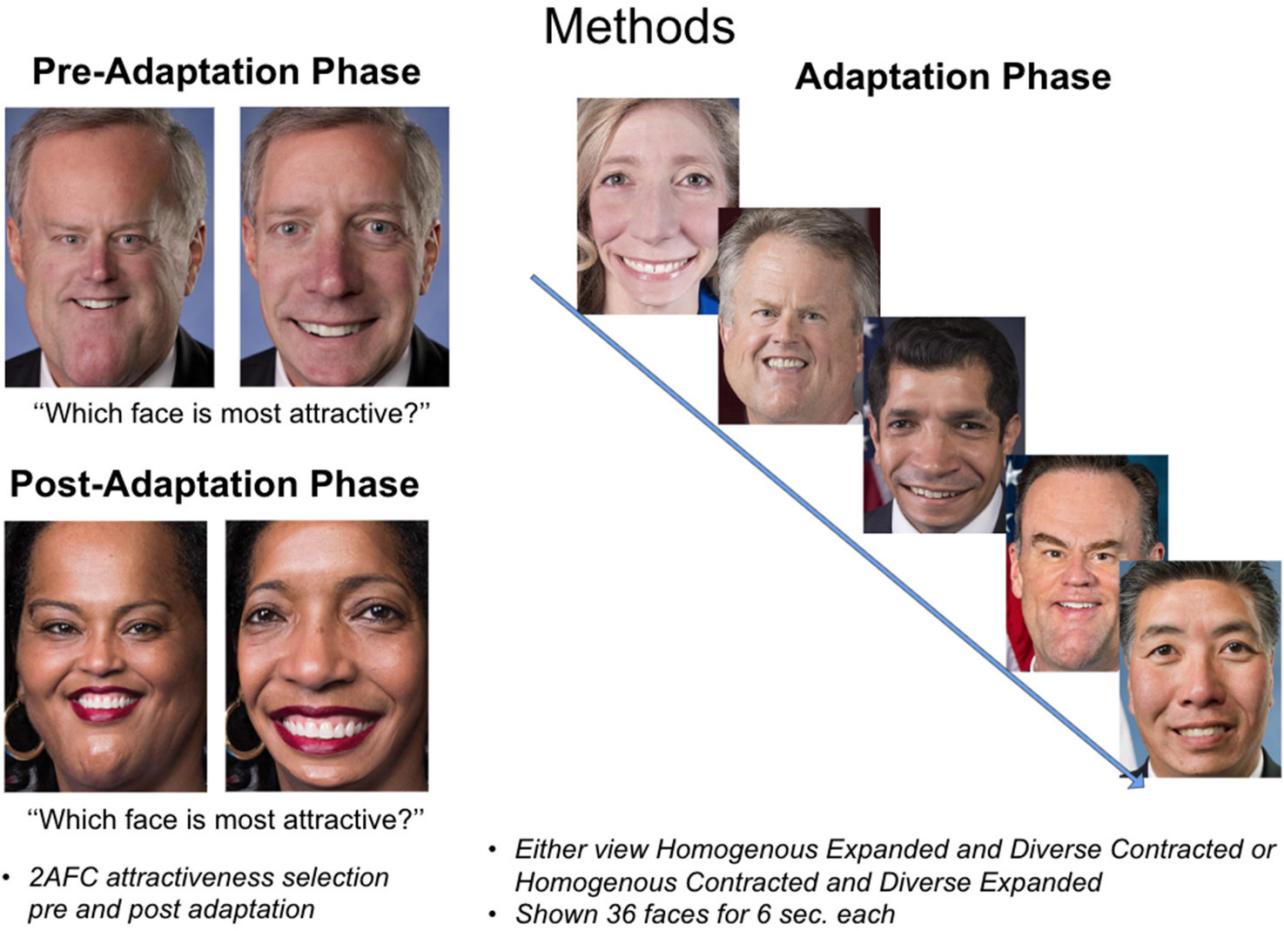
Adaptation procedure illustration. All participants underwent the same procedure phases: pre-adaptation, adaptation, and post-adaptation. The direction of the stimuli sets distortion was the only procedural change across groups (e.g., homogenous expanded/diverse contracted or vice versa).

The post-adaptation phase was almost identical to the pre-adaptation phase except that top-up faces were presented in between each face pair, and face pairs were presented in a newly randomized order, the faces were presented for 1 s each. The top-up faces were intended to maintain the adaptation throughout the post-adaptation phase and were the same faces that the participant saw in the adaptation phase but without audio clips. See Figure 2 for a visualization of the adaptation trial procedure. All participants completed demographics and questionnaires after the completion of the experimental adaptation phases (see Table 1).

**Table 1.** Demographics and political questionnaire information.

Question	Participants (*N* = 64)	Percentage (%)
Sex		
Males	30	47
Females	34	53
Age		
Average	18.32	
Standard deviation	2.20	
Range	17–35	
Average knowledge of Canadian politics	2.06	
Average knowledge of American politics	2.00	
Do you typically vote?		
Yes	40	62
No	9	14
Too young to vote	15	23
Canadian political party that most closely encompasses your values		
Liberal	24	37
NDP	16	25
Conservative	12	18
Green	1	2
I don’t know or blank	11	17
American political party that most closely encompasses your values		
Democratic	30	47
Republican	10	16
I don’t know or blank	24	38

### Demographics and Questionnaires

Following the experimental phases, participants were asked to fill out a demographic questionnaire. The questionnaire asked their age, sex, ethnicity, citizenship, whether they recognized any of the faces, which party they typically vote for in Canada, which party they would vote for if they could in the United States, and how closely they follow American and Canadian politics, whether they typically vote, and who they vote for. The question of how closely participants follow American and Canadian politics was used to measure their knowledge and to determine any notable differences between the two participant groups.

### Expected Results

Opposing aftereffects are measured when the change in the number of contracted faces selected differs within-subject across the two groups in the training set (e.g., diverse versus homogenous) and this contrast differs between the participant’s samples who have been trained on photo sets manipulated differently (homogeneous expanded and diverse contracted training vs. homogeneous contracted and diverse expanded training). For example, for the participants who adapt to contracted homogenous and expanded diverse faces, it is expected that post-adaptation these participants should prefer contracted homogenous faces more than pre-adaptation and contracted diverse faces less. If participants do in fact develop opposing aftereffects, then there should be measurable differences in preferences for contracted faces across the two training groups.

For participants who adapt to expanded homogenous and contracted diverse faces, the results would be expected to be in the opposite direction. They would be expected to prefer contracted diverse faces more than pre-adaptation and contracted homogenous faces less. If participants do in fact adapt accordingly in the expected direction, then the change in attractiveness from pre- to-post-adaptation should result in significant differences in preferences for contracted faces for homogenous and diverse faces, in this expected direction.

## Results

Participants were randomly assigned to one of two conditions: (1) contracted homogenous and expanded diverse or (2) expanded homogenous and contracted diverse. Sixty-five participants took part in the experiment. One participant was not included due to a computer error resulting in incomplete data collection. Sixty-four participants were included in the analyses. The final sample consisted of 30 males and 34 females with a mean age of 18.32 years (range: 17–35, *SD* =  2.20). Participants received partial course credit. The sample size was selected based on previous aftereffects studies indicating that approximately 30 participants per adaptation condition are sufficient in exploring opposing aftereffects (Bestelmeyer et al., 2008, 2010; Little et al., 2008; Short & Mondloch, 2010).

Before the main analyses, we examined whether the participants in the two adaptation conditions differed by how closely they followed American and Canadian politics. Participants’ average self-rated knowledge of Canadian politics was 2.06, and 2.00 for American politics on a scale from 1 to 5, with 5 being the most knowledgeable. On the question “How closely do you follow Canadian politics on a scale from 1 to 5?” a Bayes factor could not distinguish the hypothesis that the two groups of participants differed from the hypothesis that they did not (*BF*  =  3.84, 95% CI  =  [−.44, .54]). On the question “How closely do you follow American politics on a scale from 1 to 5?” a Bayes factor could not distinguish the hypothesis that the two groups of participants differed from the hypothesis that they did not (*BF*  =  2.15, 95% CI  =  [−.20, .80]).

**Figure 3.**
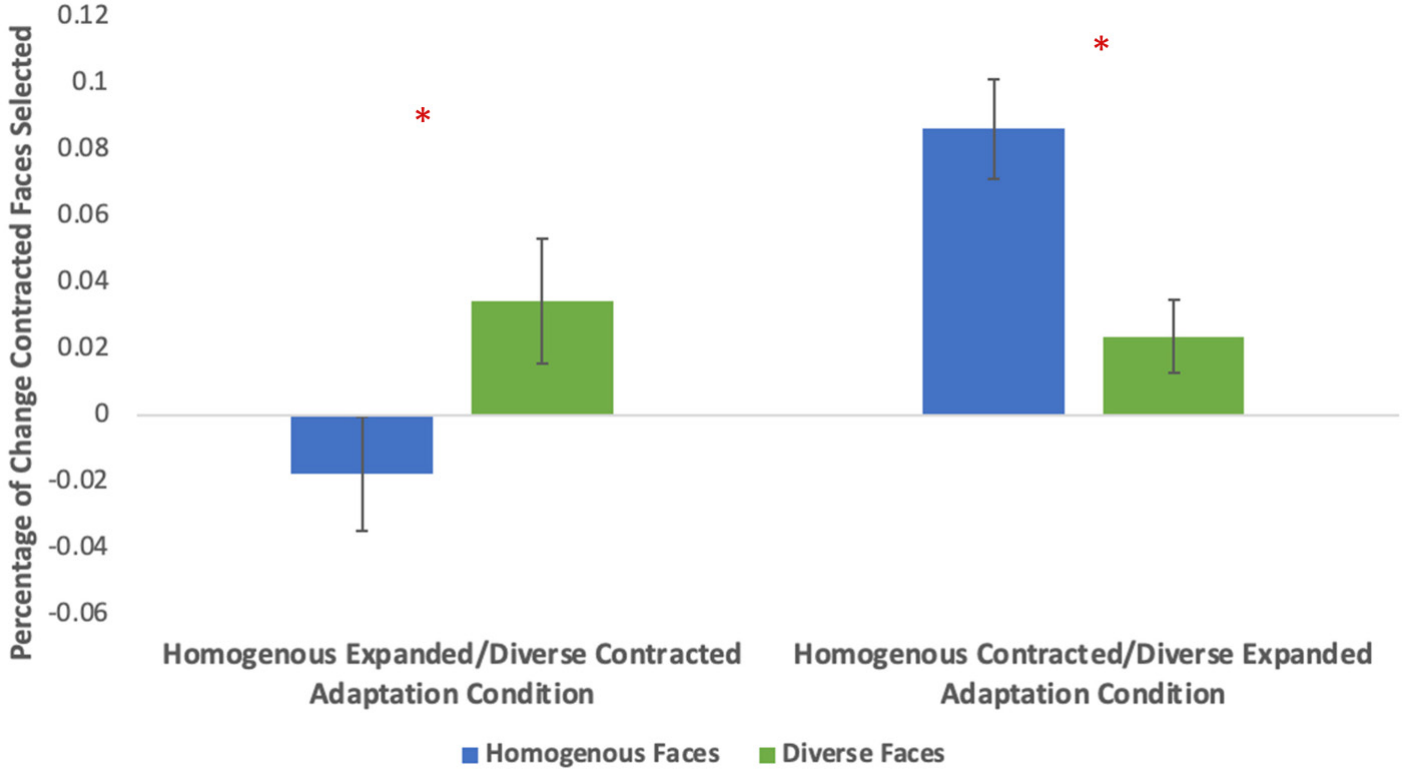
Mean change in percentage of contracted faces selected from pre to post-adaptation for each condition. There were significant differences in the change of contracted faces selected for the homogenous and diverse faces selected for both adaptation conditions.

**Figure 4.**
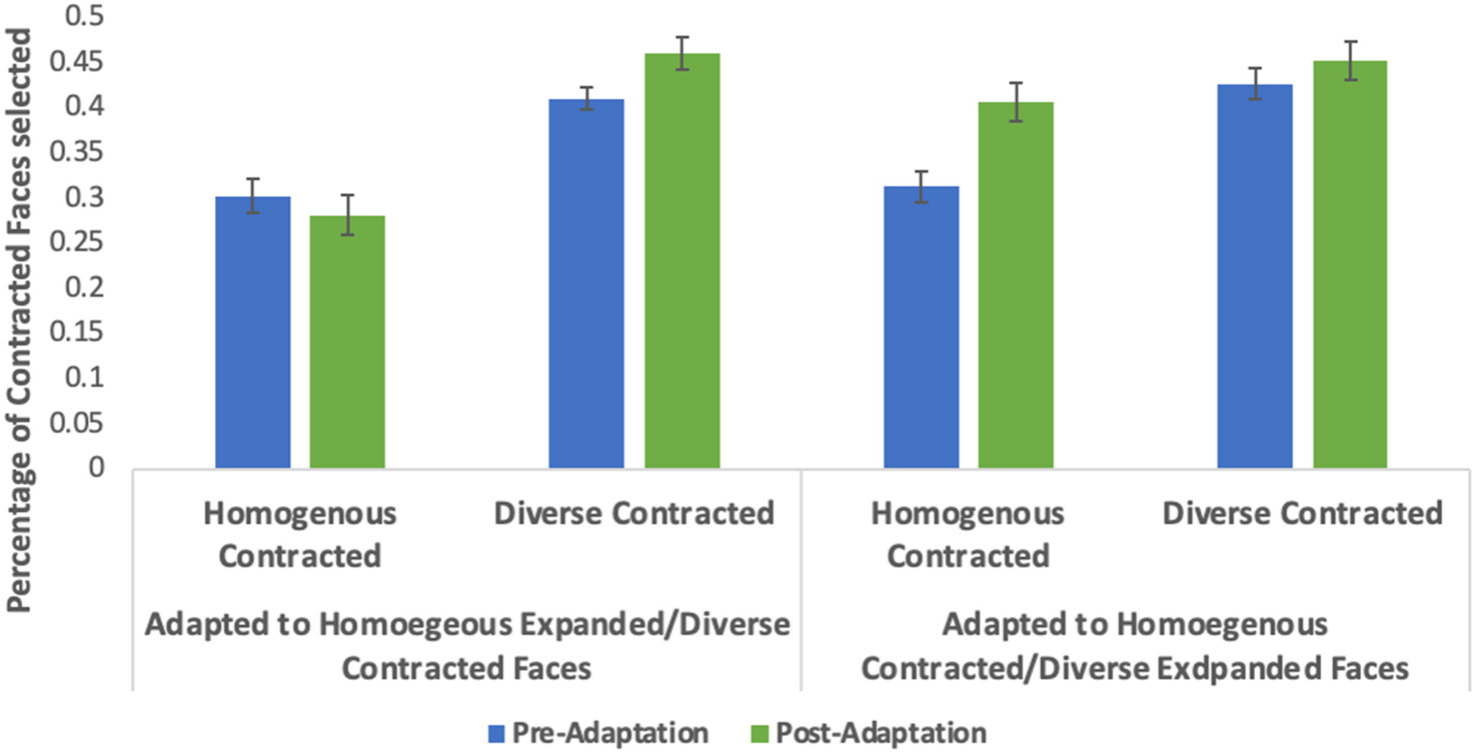
Percentage of contracted faces selected pre- and post-adaptation for both adaptation conditions. Over a total of 64 trials pre-adaptation and 64 trials post-adaptation, participants had a 50% choice of selecting a contracted face as on each trial two faces of the same individual were displayed, one 10% expanded and one 10% contract side by side.

For the main analyses, adaptation was quantified using each participant's change score, calculated by subtracting the number of contracted faces selected post-adaptation from the number of contracted faces selected pre-adaptation. A 2 (adaptation condition: adapted to expanded homogenous faces/contracted diverse or contracted homogenous/expanded diverse) by 2 (face set: homogenous or diverse test face) mixed ANOVA was conducted. Adaptation condition was the between subjects’ factor and face set within subjects’ factor. A significant interaction was found between adaptation condition and face set (*F*[1,62]  =  24.045, *p* < .001, *η_p_*^2^  =  0.279). A significant main effect for adaptation condition was found (*F*[1,62]  =  5.944, *p*  =  .018, *η_p_*^2^  =  0.087). No main effect of face set was observed (*F*[1,62]  =  0.208, *p*  =  .650). See Figure 3 for visualization of the percentage of change in selections of contracted faces from pre-adaptation to post-adaptation. See Figure 4 for a percentage of contracted faces selected pre- and post-adaptation. For the complete set of pre- and post-adaptation selections of contracted faces see the Data repository.

Planned, paired *t*-tests were used to test for adaptation for each condition independently. For the expanded homogenous/contracted heterogeneous adaptation condition significant aftereffects were observed, with significant differences between preference for diverse faces (*M*  =  1.72, *SD*  =  5.34), and homogenous faces (*M*  =  −0.88, *SD*  =  4.89), (*t*[31]  =  −2.551, *p*  =  .016). For the contracted homogenous/expanded heterogeneous adaptation condition significant aftereffects were also observed, with significant differences between preference for homogenous faces (*M*  =  4.31, *SD*  =  4.25) and diverse faces (*M*  =  1.19, *SD*  =  3.09), (*t*[31]  =  5.47, *p* < .001). Both conditions were consistent with adaptation (see Figure 3).

## Discussion

The purpose of the current study was to test (1) whether a face set that is diverse with respect to race and gender could support a face aftereffect and (2) whether race and gender diversity is the kind of socially meaningful cue to category membership that is thought necessary for the creation of opposing aftereffects. Participants adapted to a homogenous face set consisting of only White middle-aged male faces and a diverse face set consisting of a balanced sample of White, Asian, Black, and Latino male and female faces while the two face sets were distorted in the opposite direction.

First, aftereffects were observed, including aftereffects produced by adaptation to the diverse face set. These results indicate that though there was physical variation across the faces in the diverse face set, the adaptation phase of the experiment produced a coherent directional adaptation. This is the first indication that it is possible to adapt to a face set that has a physical variation with respect to sex and gender.

Second, opposing aftereffects were found between the diverse and homogenous face sets. This is the first indication that the diversity of a face set might be the kind of socially meaningful cue to group membership that is necessary for the formation of opposing aftereffects. This is the first evidence that diversity itself may be a cue to social category membership.

The diverse face set in the current study consisted of a balanced sample of four different races and both male and female faces. Typically, during the opposing aftereffects paradigm, the physical variation within a face set only differs based on the individual's identities, while their race or gender is the same within the face set but different between the face sets (Armann et al., 2011; Jaquet et al., 2008; Little et al., 2008). Recent research on ensemble coding suggests that when perceiving groups of faces it is possible for individuals to extract uniquely social properties from groups of people, such as diversity and hierarchy even with minimal social cues (Phillips et al., 2018). Not only does the current study suggest that the diversity of faces could be a cue to social category membership, but also that it is possible to adapt to a group of faces that vary physically in a number of ways.

The ability of individuals to perceive the diverse face set as a group is remarkable considering how physically different the faces were. Zellmer-Bruhn et al. (2008) suggest that when a new group of faces is presented the most obvious social categories, such as sex, race, and ethnicity, will shape the perception of similarity and diversity within groups. The more distinct the faces are, the greater the possibility of viewing one group of faces based on multiple sub-categories face groups (Lau & Murnighan, 1998). However, when diverse identities are perceived in a more complex and integrated manner it is possible that the perceiver is more likely to perceive the group as one diverse whole rather than separate identities (Roccas & Brewer, 2002). It is possible that during adaptation the diverse face set may have facilitated a more complex perception of the diverse set, such that the diverse face set was viewed as a group, separate from the homogenous set.

The ability to adapt to multiple races and genders at the same time is also consistent with one previous study's findings that it is possible to adapt to the expression of a face set and the gender at the same time as one face set, using an opposing aftereffects paradigm (Bestelmeyer et al., 2010). The results from the current study expand on this phenomenon by exploring whether differing races and sexes could also be adapted to as one face set. As opposing aftereffects were observed between the homogenous and diverse face set it is possible that the diverse face set was adapted to as one group due to the ability to adapt to multiple different sexes and races independently in the same direction at once (Bestelmeyer et al., 2010; Bruce & Young, 1986) and perceiving the diverse group as one ensemble (Haberman & Whitney, 2009).

As this is the first evidence of adaptation to a face set that varies by both gender and race there are multiple interpretations of how this effects the norm-based coding of these faces. First, it is possible that while adapting to the diverse face set, multiple face templates (four races by two genders) were adapted to simultaneously as there are separate face templates by gender and race (Jaquet & Rhodes, 2008; Jaquet et al., 2008; Rhodes et al., 2004; Little et al., 2008). Under this interpretation, it is possible that when adapting to the diverse face sets each of the presented genders and races were adapted to in the same direction due to those specific templates all being adapted to at once.

Second, it is possible that a broader umbrella representation of all these faces underlies the perception of the entire face set, and that neural representation is what was adapted. Although aftereffects evidence indicates that there are multiple separate templates for differing genders and races, evidence on ensemble coding suggests that when the visual system is presented with a several faces rather than coding every individual element presented it favors a summary or average (Haberman & Whitney, 2009). For example, when perceiving an ensemble of faces, participants are able to detect average facial morphology (de Fockert & Wolfenstein, 2009), emotional expression (Haberman & Whitney, 2009; Won & Jiang, 2013), and attractiveness (Walker & Vul, 2014). Therefore, it is possible that a summary perception represents the diverse faces. Under this interpretation, an umbrella or average template may have facilitated the formation of aftereffects in the diverse face set.

The second hypothesis examined whether race and gender diversity are a socially meaningful cue to category membership. In order to evoke opposing aftereffects, it is thought that two face sets must be both physically distinct (Bestelmeyer et al., 2008) and socially meaningful (Short & Mondloch, 2010). In the current study, the opposing aftereffects speak to the psychological grouping that underlies category membership. The social significance of diversity was a relevant cue to social group membership that may support opposing aftereffects. Diversity has been found to be a relevant social cue that can be extracted when viewing an ensemble of faces (Phillips et al., 2018). In the United States, the Democratic party is increasingly more racially diverse than the Republican party (Newport, 2013), which results in diversity itself being a cue to party membership. The fact that opposing aftereffects were observed suggests that diversity was a socially meaningful cue that influenced category membership perception.

### Limitations and Future Directions

One limitation of our current methodology is the use of “attractiveness” as a proxy to examine normality. Several studies have used attractiveness judgments in the past to evaluate normality throughout categorical opposing aftereffects paradigms (Foglia et al., 2021; Little et al., 2005; Short et al., 2011; Short & Mondloch, 2015). However, there is some evidence that the norm is not the pinnacle of attractiveness (e.g., Brown & Slaughter, 2011; Winkler & Rhodes, 2005). Due to this, some other studies on opposing aftereffects have chosen to use the wording of judging the “normality” of faces presented, rather than “attractiveness” because of this (Jaquet et al., 2008, Little et al., 2008). Given the context in which the face sets were presented in the current study, it is possible that the perceived attractiveness of faces may be influenced by participants’ political ideologies. Asking instead for a perceived “normality” may decrease this potential confound. Future studies should consider which form of wording and methodology may best suit evaluating the face sets and categories of interest, and the effect this could potentially have on their results.

Secondly, in the current study significant opposing aftereffects were observed in both adaptation conditions, though in one of the two conditions participants showed evidence of their adaptation to the homogenous contracted faces in their perception of diverse faces. While we and others would still take this as evidence of opposing aftereffects (see Short et al. (2011) and there is a significant difference between two face groups, one face group is more consistently adapted to in the expected direction of an opposing aftereffect. We suspect that this relates to the peculiar relationship between our two face image sets: they overlap. While other tests of opposing aftereffects used completely distinct face sets (e.g., male and female or Caucasian and Chinese, etc.) our Homogenous face set consisted entirely of middle-aged White males, whereas our Diverse face set included White as well as Latino, Black, and Asian faces. Thus, it is not surprising that those who adapted to Contracted middle-aged White male faces and were then tested with face stimuli that included that group may have exhibited some influence of the adaptation they experienced.

We found that in order to induce the strongest adaptation to a diverse group of faces, contracted faces are better adaptors than expanded. This is not the first time that contracted faces have been found to be better adaptors than expanded during opposing aftereffects paradigms. Previously, Walsh et al. (2015) reported a differing pattern of adaptation effects following adaptation to contracted versus expanded faces. Participants who adapted to contracted faces showed larger aftereffects that those who adapted to expanded faces. It is possible that contracted faces are not perceptually equivalent to expanded, even if the distortions are physically equated.

As the results from the current study provide insight into the ability to adapt to a diverse face set as one coherent group. Future studies might test whether this finding is generalized across other groupings of race and sex. For example, were the results of the current study unique to a homogenous face category that consisted of White middle-aged males? Would similar results occur between homogenous and diverse face sets if a minority group was chosen as the homogenous social category? It is possible that social hierarchy may play a role in determining which groups lack diversity. Perhaps changing the homogenous group to another race or sex would lead to null results. Further research is necessary in order to understand how diversity and social hierarchy impact visual adaptation to faces.

## Conclusion

The purpose of this study was to test (1) whether adapting to a diverse face set could lead to a coherent aftereffect and (2) whether it is possible to adapt to a homogenous and a diverse face set simultaneously using an opposing aftereffect paradigm. Results revealed that opposing aftereffects were evoked for the homogenous and diverse face sets. These results suggest that it is possible to adapt to a set of faces that differs physically within the set based on sex and race while also adapting to another set that does not. This was the first evidence that diversity may be a cue to social group membership sufficient to support opposing aftereffects.

## **Appendix A.** Names Demographic information of the Members of Congress used as stimuli.

**Table:**

Name	Race	Gender	Political Party	Phase
Ben Cline	White	Male	Republican	Adaptation Adaptation Adaptation Adaptation Adaptation Adaptation Adaptation Adaptation Adaptation Adaptation Adaptation Adaptation Adaptation Adaptation Adaptation Adaptation Adaptation Adaptation Adaptation Adaptation Adaptation Adaptation Adaptation Adaptation Adaptation Adaptation Adaptation Adaptation Adaptation Adaptation Adaptation Adaptation Pre- and post-adaptation Pre- and post-adaptation Pre- and post-adaptation Pre- and post-adaptation Pre- and post-adaptation Pre- and post-adaptation Pre- and post-adaptation Pre- and post-adaptation Pre- and post-adaptation Pre- and post-adaptation Pre- and post-adaptation Pre- and post-adaptation Pre- and post-adaptation Pre- and post-adaptation Pre- and post-adaptation Pre- and post-adaptation
Bryan Steil	White	Male	Republican	
Chris Smith	White	Male	Republican	
Daniel Webster	White	Male	Republican	
Drew Ferguson	White	Male	Republican	
Jeff Fortenberry	White	Male	Republican	
Jim Banks	White	Male	Republican	
John Joyce	White	Male	Republican	
Ken Buck	White	Male	Republican	
Mark Meadows	White	Male	Republican	
Rodney Davis	White	Male	Republican	
Steve Womack	White	Male	Republican	
Tim Burchett	White	Male	Republican	
Tom Emmer	White	Male	Republican	
Mac Thornberry	White	Male	Republican	
Tom McClintock	White	Male	Republican	
Joe Neguse	Black	Male	Democrat	
John Lewis	Black	Male	Democrat	
Karen Bass	Black	Female	Democrat	
Val Demings	Black	Female	Democrat	
Doris Matsui	Asian	Female	Democrat	
Grace Meng	Asian	Female	Democrat	
Mark Takano	Asian	Male	Democrat	
Ted Lieu	Asian	Male	Democrat	
Jimmy Gomez	Latino	Male	Democrat	
Linda Sanchez	Latino	Female	Democrat	
Nanette Barragan	Latino	Female	Democrat	
Raul Ruiz	Latino	Male	Democrat	
David Trone	White	Male	Democrat	
Ed Case	White	Male	Democrat	
Sean Casten	White	Male	Democrat	
Susan Wild	White	Female	Democrat	
Alcee Hastings	Black	Male	Democrat	
Lisa Blunt Rochester	Black	Female	Democrat	
Stephanie Murphy	Asian	Female	Democrat	
TJ Cox	Asian	Male	Democrat	
Juan Vargas	Latino	Male	Democrat	
Norma Torres	Latino	Female	Democrat	
Cheri Bustos	White	Female	Democrat	
Scott Peters	White	Male	Democrat	
Brad Wenstrup	White	Male	Republican	
Bradley Byrne	White	Male	Republican	
James Comer	White	Male	Republican	
Jeff Duncan	White	Male	Republican	
Michael Guest	White	Male	Republican	
Ralph Abraham	White	Male	Republican	
Roger Marshall	White	Male	Republican	
Russ Fulcher	White	Male	Republican	
